# Development and implementation of a performance improvement project in adult intensive care units: overview of the Improving Medicine Through Pathway Assessment of Critical Therapy in Hospital-Acquired Pneumonia (IMPACT-HAP) study

**DOI:** 10.1186/cc9988

**Published:** 2011-01-25

**Authors:** Julie E Mangino, Paula Peyrani, Kimbal D Ford, Daniel H Kett, Marcus J Zervos, Verna L Welch, Ernesto G Scerpella, Julio A Ramirez, the IMPACT-HAP Study Group

**Affiliations:** 1 The Ohio State University, 410 West 10th Ave, N-1150 Doan Hall Columbus, OH 43210, USA; 2 University of Louisville, 501 E Broadway, MedCenter One Suite 380, Louisville, KY 40202, USA; 3 Pfizer Inc., 500 Arcola Road, Collegeville, PA 19426, USA; 4 University of Miami/Jackson Memorial Hospital, 1611 NW 12th Ave, Central Wing-Room 455, Miami, FL 33136, USA; 5 Henry Ford Health System/Wayne State University School of Medicine, 2799 West Grand Blvd, Detroit, MI 48202, USA

## Abstract

**Introduction:**

In 2005 the American Thoracic Society and Infectious Diseases Society of America (ATS/IDSA) published guidelines for managing hospital-acquired pneumonia (HAP), ventilator-associated pneumonia (VAP), and healthcare-associated pneumonia (HCAP). Although recommendations were evidence based, collective guidelines had not been validated in clinical practice and did not provide specific tools for local implementation. We initiated a performance improvement project designated Improving Medicine Through Pathway Assessment of Critical Therapy in Hospital-Acquired Pneumonia (IMPACT-HAP) at four academic centers in the United States. Our objectives were to develop and implement the project, and to assess compliance with quality indicators in adults admitted to intensive care units (ICUs) with HAP, VAP, or HCAP.

**Methods:**

The project was conducted in three phases over 18 consecutive months beginning 1 February 2006: 1) a three-month planning period for literature review to create the consensus pathway for managing nosocomial pneumonia in these ICUs, a data collection form, quality performance indicators, and internet-based repository; 2) a six-month implementation period for customizing ATS/IDSA guidelines into center-specific guidelines via educational forums; and 3) a nine-month post-implementation period for continuing education and data collection. Data from the first two phases were combined (pre-implementation period) and compared with data from the post-implementation period.

**Results:**

We developed a consensus pathway based on ATS/IDSA guidelines and customized it at the local level to accommodate formulary and microbiologic considerations. We implemented multimodal educational activities to teach ICU staff about the guidelines and continued education throughout post-implementation. We registered 432 patients (pre- vs post-implementation, 274 vs 158). Diagnostic criteria for nosocomial pneumonia were more likely to be met during post-implementation (247/257 (96.1%) vs 150/151 (99.3%); *P = *0.06). Similarly, empiric antibiotics were more likely to be compliant with ATS/IDSA guidelines during post-implementation (79/257 (30.7%) vs 66/151 (43.7%); *P *= 0.01), an effect that was sustained over quarterly intervals (*P *= 0.0008). Between-period differences in compliance with obtaining cultures and use of de-escalation were not statistically significant.

**Conclusions:**

Developing a multi-center performance improvement project to operationalize ATS/IDSA guidelines for HAP, VAP, and HCAP is feasible with local consensus pathway directives for implementation and with quality indicators for monitoring compliance with guidelines.

## Introduction

The American Thoracic Society and Infectious Diseases Society of America (ATS/IDSA) published guidelines for the management of hospital-acquired pneumonia (HAP), ventilator-associated pneumonia (VAP), and healthcare-associated pneumonia (HCAP) in 2005 [1]. The guidelines emphasize several major principles. First, HAP, VAP, and HCAP should be treated promptly and adequately because patients who experience delays in appropriate therapy have increased mortality. Second, local microbiologic data should be used to customize management guidelines within centers. Third, excessive antibiotic use should be avoided by accurately diagnosing the infection, using culture results to de-escalate initial therapy, and minimizing the duration of therapy. Fourth, prevention strategies based on modifiable risk factors for HAP should be implemented.

The goal of the ATS/IDSA guidelines is to provide an organizational framework for initial evaluation and management of adults with bacterial HAP, VAP, or HCAP; however, the authors acknowledged that the guidelines had several limitations [1]. For example, individual recommendations were based on the best available evidence, but the impact of the collective guidelines on clinical outcome had not been validated. Similarly, the guidelines provide algorithms and management strategies, but they do not provide specific tools for implementation at the local level.

Inspired by the ATS/IDSA guidelines [1] and the desire to assess and improve outcomes in adults with HAP in the intensive care unit (ICU), we initiated a performance improvement project designated Improving Medicine through Pathway Assessment of Critical Therapy in Hospital-Acquired Pneumonia (IMPACT-HAP). Our objectives were to develop, implement, and assess this performance improvement project. To assess compliance with management guidelines, we developed a series of quality indicators and herein report our findings before and after implementation of this performance improvement project. Preliminary findings have been previously reported [2-4].

## Materials and methods

### Participants

We conducted a multicenter performance improvement project at four academic tertiary care centers in the United States. Participating centers were The Ohio State University Medical Center, Columbus, Ohio; Henry Ford Health System, Detroit, Michigan; University of Miami Miller School of Medicine/Jackson Memorial Hospital, Miami, Florida; and University of Louisville, Louisville, Kentucky. The project was conducted in selected adult medical ICUs at two centers (Columbus, 57 participating ICU beds and Miami, 18 beds) on the basis of local staffing to conduct the project; and in all adult ICUs at two centers (Louisville, 61 beds and Detroit, 144 beds). The project was approved by the institutional review board at each participating center; each waived the need for informed consent.

### Development of the performance improvement project and educational efforts

We conducted a prospective performance improvement project in three phases over 18 consecutive months from 1 February 2006 through 31 July 2007. The first phase was a three-month pre-implementation period. Representatives from each center planned the project. The IMPACT-HAP investigators evaluated the ATS/IDSA guidelines [1] and reviewed the clinical literature to develop a consensus pathway for the management of HAP, VAP, and HCAP for ICU patients. We then defined a series of quality performance indicators to assess compliance with management guidelines. We created a form to collect patient-level data (demographics, laboratory, treatment, outcomes) and an Internet-based repository to transfer the data to the IMPACT-HAP study center at the University of Louisville.

The second phase was a six-month implementation period. Principal investigators at each center formed multidisciplinary teams to customize ATS/IDSA guidelines and create a local consensus pathway based on their respective order sets, hospital formulary, and local epidemiology (unit-specific antibiograms), including consideration of center-specific resistance patterns. Educational efforts were initiated during implementation. The third phase was a nine-month post-implementation period with continued education and data collection.

### Patient inclusion criteria and assessment

Adults in participating ICUs were eligible for inclusion in the database if they met ATS/IDSA definitions for HAP, VAP, or HCAP [1], including clinical suspicion of evolving pneumonia while in the ICU, with new or progressive infiltrates on chest radiograph and at least two of the following: new or increased cough, sputum production, tracheal secretions, or shortness of breath; fever or hypothermia; leukocytosis, left shift, or leukopenia; or deterioration of pulmonary function [5]. Patients were followed until hospital discharge, death, or Day 28, whichever occurred first.

Comorbid conditions were prospectively defined and extracted from patient records. Respiratory disease was defined as a history of chronic obstructive pulmonary disease. Renal disease was defined as a history of chronic renal disease, or abnormal blood urea nitrogen and creatinine. Cardiac disease was defined as systolic or diastolic ventricular dysfunction by history, physical examination, chest radiogram, or echocardiogram. Immunosuppression was defined as active malignancy; AIDS; end-stage renal, liver, or lung disease; steroids (prednisone ≥10 mg for >7 days); or active chemotherapy or radiotherapy within 30 days. Severe sepsis was determined by calculation of the sepsis criteria and organ dysfunction criteria [6], which were abstracted from the chart at the time of enrollment or on Day 0.

Investigators completed a data collection form for each patient. Principal investigators at each site reviewed each form, added any missing data, and internally validated the information before transferring it via the Internet to the repository. Validation of data quality was also performed at the IMPACT-HAP study center. Initial empiric therapy was classified as appropriate if the isolated pathogen was susceptible to at least one prescribed antibiotic. For the purpose of this analysis, data from the first two phases were combined into one nine-month period (that is, pre-implementation and implementation (hereafter, pre-implementation period)) and compared with data from the third nine-month phase (that is, post-implementation period).

Selection of empiric antibiotics was based on ATS/IDSA guidelines [1] and the presence of risk factors for multidrug-resistant organisms (MDROs). For example, therapy was considered guideline compliant if, within one day of pneumonia onset, patients with risk factors for MDROs received dual gram-negative therapy plus either linezolid or vancomycin for methicillin-resistant *Staphylococcus aureus *(MRSA). Reasons for noncompliance were recorded. Clinical outcome was categorized on Day 14 as cure (complete resolution of signs and symptoms of pneumonia), improvement (partial resolution), or failure (deterioration of signs and symptoms of pneumonia). Clinical success was defined as cure or improvement.

### Statistics

Descriptive statistics were calculated for baseline demographics and severity-of-illness scores, compliance with guidelines, and reasons for noncompliance in patients enrolled during the pre-implementation and post-implementation periods. Compliance with each quality performance indicator was calculated as the percentage of patients who met the criteria for each indicator based on the total number evaluable for each quality indicator. Between-implementation differences were compared using the Chi-square test or, if applicable, Fisher's exact test for categorical variables and, for continuous variables, the Student's *t*-test for normally distributed variables and the Wilcoxon rank sum test for non-normally distributed variables. *P-*values of ≤0.05 were considered to be statistically significant. All data analyses were performed using SAS software, version 9.2 (SAS Institute Inc., 100 SAS Campus Drive, Cary, NC 27513-2414, USA).

## Results

### Consensus pathway

**Figure 1:**
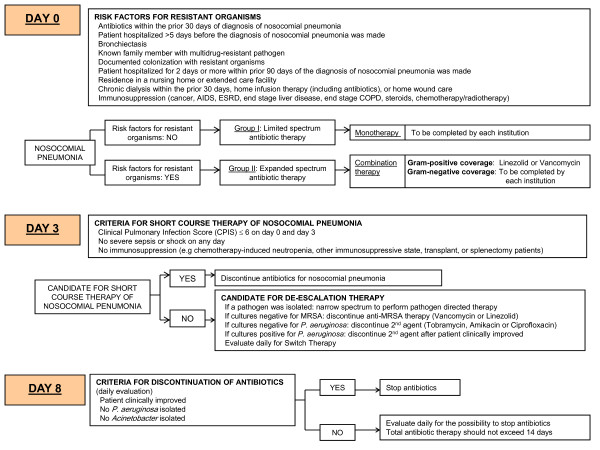
**IMPACT-HAP consensus pathway for the management of nosocomial pneumonia in the intensive care unit **.

A consensus pathway was developed based on the principles of the ATS/IDSA guidelines [1] for the management of adults with HAP, VAP, and HCAP in the ICU (Figure 1). Principal investigators, clinical pharmacists and infection control practitioners from four academic medical centers participated in three teleconferences and a face-to-face consensus meeting. Principal investigators had monthly teleconferences throughout the project to maintain consensus; study coordinators had teleconferences every two months.

On Day 0, patients with suspected nosocomial pneumonia (on the basis of the recognition of the presenting signs and symptoms) were stratified by the presence or absence of risk factors for MDROs. Patients without risk factors for MDROs were to receive limited-spectrum monotherapy, which included the following options: ceftriaxone, fluoroquinolone, ampicillin/sulbactam, or ertapenem. Most centers chose ceftriaxone or moxifloxacin, except one center chose ampicillin/sulbactam or ceftriaxone plus azithromycin or moxifloxacin.

Patients with risk factors for MDROs were to receive expanded-spectrum combination therapy with dual gram-negative coverage and either linezolid or vancomycin as anti-MRSA therapy. Of the ATS/IDSA options for primary gram-negative coverage (antipseudomonal cephalosporin, antipseudomonal carbapenem, or β-lactam/β-lactamase inhibitor), centers chose the following three options: cefepime, imipenem or piperacillin-tazobactam. Of the ATS/IDSA options for secondary gram-negative coverage (antipseudomonal fluoroquinolone or aminoglycoside), most centers chose tobramycin or amikacin.

The intent of the IMPACT-HAP pathway (Figure 1) was to assist clinicians at the participating ICUs in recognizing the signs and symptoms of suspected nosocomial pneumonia and subsequently delivering antibiotics compliant with the ATS/IDSA guidelines as quickly as possible. Centers were permitted to adapt the consensus pathway based on their formulary and local susceptibility data determined by unit-specific antibiograms. Two centers had, as part of their educational tools, secondary gram-negative therapy for all patients with risk factors for MDROs and suspected HAP, VAP, or HCAP (see example in Additional file 1). Two centers reserved secondary gram-negative therapy for VAP only, on the basis of their local microbiology and the low risk of resistant gram-negative pathogens in patients not requiring mechanical ventilation. Although not in the ATS/IDSA guidelines, colistin was prospectively deemed an acceptable secondary gram-negative agent when carbapenem-resistant *Acinetobacter *was of concern, as was the case in one ICU.

On Day 3, antibiotic therapy was to be discontinued in patients who met the criteria for short-course therapy as described by Singh and colleagues [7]. Criteria for short-course therapy were clinical pulmonary infection score (CPIS) of ≤6 on days 0 and 3, and no sepsis, shock, or immunosuppression. De-escalation was to be considered on Day 3 if patients were not candidates for short-course therapy. Criteria for de-escalation were clinical improvement and cultures positive for a pathogen that allowed antibiotics to be narrowed or focused based on susceptibility. On Day 8, antibiotic therapy was to be discontinued if the patient had improved clinically and did not have cultures positive for *Pseudomonas aeruginosa *or *Acinetobacter *spp. The total duration of antibiotics was not to exceed 14 days.

### Implementation of the performance improvement project

We developed a data collection form to collect patient demographics, comorbidities, physical examination, laboratory, and chest radiograph findings; risk factors for MDROs; and six quality performance indicators. We tested the data collection form and data repository in the first 30 patients, adjusted the form, and then used the revised form for subsequent patients.

Quality performance indicators were expressed as percentages and calculated by dividing the number of patients who met the criteria for the quality indicator by the total number of patients in whom antibiotics for HAP, VAP, or HCAP were started, unless otherwise stated. The numerator for the first quality indicator (QI-1) was the number of patients who met diagnostic criteria for HAP, VAP, or HCAP and whose physicians recognized and appropriately documented the risk factors for HAP, VAP, or HCAP. The numerator for QI-2 was the number of patients in whom respiratory (QI-2a) and blood cultures (QI-2b) were obtained *before *antibiotics were initiated. The numerator for QI-3 was the number who received antibiotics compliant with ATS/IDSA guidelines [1]. QI-4 was calculated by dividing the number of patients who received short-course therapy by the total who were candidates for short-course therapy. QI-5 was calculated by dividing the number who underwent de-escalation on Day 3 by the total who were candidates for de-escalation. QI-6 was defined by dividing the number of patients with clinical success at end of therapy or, if the patient remained on antibiotics, on Day 14 by the total who were evaluable for clinical outcome.

Educational efforts were started during the implementation phase. The principal investigators led a variety of didactic forums including grand rounds, internal medicine house staff lectures, infectious diseases and pulmonary and critical care divisional conferences, ICU-specific physician and nursing staff unit meetings, and pharmacy and respiratory therapy conferences -- all emphasizing the ATS/IDSA guidelines [1] with the center-specific management plan. We used multimodal strategies, which included lectures utilizing a standardized slide set, posters within the ICUs and patient cubicles, emails to new ICU monthly attending physicians and pulmonary fellows containing the center-specific flyer that could be folded into pocket-sized references, and multiple personal interactions. Education was continued informally throughout the post-implementation period primarily to target the newly rotating house staff.

### Patients

A total of 449 patients were captured in the Internet-based repository; 17 were excluded because of missing data. Of the remaining 432 patients, 274 were registered during pre-implementation (1 February through 31 October 2006), and 158 during post-implementation (1 November 2006 through 31 July 2007). Some differences existed in patient profiles when grouped by time of registry (Table 1). A higher percentage of white patients were registered during pre-implementation (pre- vs post-implementation, 65.8% vs 57.6%; *P *= 0.04 for race). Distribution of comorbidities was similar, except that fewer patients had renal disease during pre-implementation (15% vs 24.5%; *P *= 0.02). CPIS at baseline and presence of severe sepsis were similar during the two periods, but Acute Physiology and Chronic Health Evaluation (APACHE) II score was lower during pre-implementation (mean, 20.1 vs 21.6; *P *= 0.03). Patients were also less likely to have a risk factor for an MDRO during pre-implementation (92.6% vs 98.0%; *P *= 0.02).

**Table 1:** Baseline demographics and severity of illness in patients with HAP, VAP, and HCAP in the ICU, stratified by enrollment period

Characteristic	Number/number evaluable (%), unless otherwise indicated		*P-*value
	Pre-implementation (*n *= 274)	Post-implementation (*n *= 158)	
Age in years, mean ± SD	57.1 ± 17.1	59.4 ± 16.8	0.20
Age <65 years	167/257 (65.0)	89/151 (58.9)	0.22
Male gender	173/257 (67.3)	93/151 (61.6)	0.24
Race			0.04
White	169/257 (65.8)	87/151 (57.6)	
Black	79/257 (30.7)	50/151 (33.1)	
Other	9/257 (3.5)	14/151 (9.3)	
Weight in pounds, mean ± SD	182.7 ± 62.8	180.0 ± 65.5	0.68
Comorbid conditions			
Respiratory	58/255 (22.8)	32/150 (21.3)	0.74
Renal	38/253 (15.0)	37/151 (24.5)	0.02
Cardiac	52/255 (20.4)	36/151 (23.8)	0.42
Malignancy	41/256 (16.0)	20/150 (13.3)	0.47
Immunosuppression^a^	93/257 (36.2)	65/151 (43.0)	0.17
Severity of illness scores			
APACHE II score, mean ± SD	20.1 ± 7.0	21.6 ± 7.7	0.03
CPIS, mean ± SD	6.2 ± 1.9	6.3 ± 1.7	0.59
Presence of severe sepsis	206/274 (75.2)	127/157 (80.9)	0.17
Risk factor for multidrug resistant pathogen^b^			
Any	238/257 (92.6)	148/151 (98.0)	0.02
Antibiotic within 30 days	154/236 (65.3)	90/148 (60.8)	0.55
Hospitalized ≥5 days before HAP antibiotics	166/238 (69.8)	79/148 (53.4)	0.001
Hospitalized ≥2 days within 90 days	92/238 (38.7)	81/148 (54.7)	0.01
Residence in nursing home or extended care	35/238 (14.7)	33/147 (22.5)	0.02
Chronic dialysis within 30 days	13/238 (5.5)	11/145 (7.6)	0.05

APACHE II, Acute Physiology and Chronic Health Evaluation II; CPIS, Clinical Pulmonary Infection Score; SD, Standard Deviation.

^a ^Immunosuppression, active malignancy; AIDS; end-stage renal, liver, or lung disease; steroids (prednisone ≥10 mg for >7 days); or active chemotherapy or radiotherapy within 30 days. ^b ^The three most common risk factors and those with significant between-group differences are listed.

### Quality performance indicators

**Figure 2:**
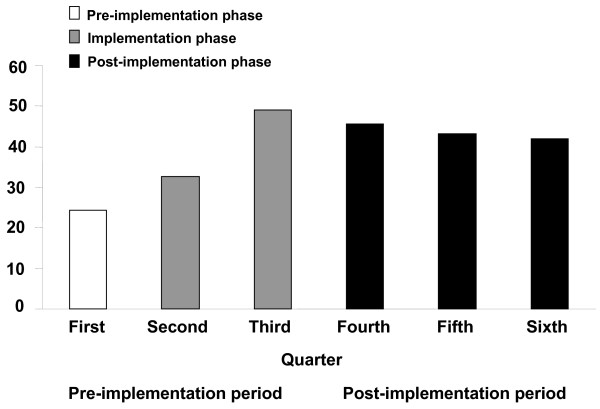
**Percentage of patients receiving initial empiric therapy compliant with ATS/IDSA guidelines at quarterly intervals **. *P *= 0.0008 for trend over time.

Evaluation of compliance with quality performance indicators revealed some differences between periods (Table 2). Specifically, empiric antibiotics were more likely to be compliant during post-implementation with ATS/IDSA guidelines (QI-3: pre- vs post-implementation, 30.7% vs 43.7%; *P *= 0.01) and with center-specific guidelines (35.8% vs 51.0%; *P *= 0.002). Analysis of QI-3 at quarterly intervals revealed improved compliance with ATS/IDSA guidelines over time (*P *= 0.0008 for trend over time; Figure 2). The most common reason for noncompliance with ATS/IDSA guidelines during both periods was failure to use a secondary gram-negative agent (82/132 (62.1%) vs 56/71 (78.9%)). Compliance with short-course therapy doubled during post-implementation, but this improvement was not statistically significant (25.0% vs 52.9%; *P *= 0.10) and only a small number of patients (*n *= 37) were eligible for short-course therapy during the project. Other between-period differences in compliance with quality indicators were not statistically significant.

**Table 2:** Quality indicators during pre- and post-implementation of consensus pathway for managing pneumonia in the ICU

Quality indicator	**Number/number evaluable**^**a **^**(%)**		*P-*value
	Pre-implementation (*n *= 274)	Post-implementation (*n *= 158)	
QI-1: Diagnostic criteria for HAP, VAP or HCAP met	247/257 (96.1)	150/151 (99.3)	0.06
QI-2a: Respiratory sample obtained before antibiotics	253/265 (95.5)	134/141 (95.0)	0.81
QI-2b: Blood culture obtained before antibiotics	214/264 (81.1)	121/141 (85.8)	0.23
QI-3: Empiric therapy compliant with ATS/IDSA guidelines [1]^b^	79/257 (30.7)	66/151 (43.7)	0.01
QI-4: Short-course therapy performed	5/20 (25.0)	9/17 (52.9)	0.10
QI-5a: De-escalation possible	173/266 (65.0)	96/140 (68.6)	0.47
QI-5b: De-escalation possible and performed	56/173 (32.4)	36/96 (37.5)	0.40
QI-6: Clinical success at day 14	170/250 (68.0)	89/134 (66.4)	0.75

ATS, American Thoracic Society; HAP, hospital-acquired pneumonia; HCAP, healthcare-associated pneumonia; ICU, intensive care unit; IDSA, Infectious Diseases Society of America; VAP, ventilator-associated pneumonia.

^a ^All patients were not evaluable for every quality indicator. ^b ^Empiric therapy compliant with center-specific guidelines: pre- vs post-implementation, 92/257 (35.8%) vs 77/151 (51.0%); *P *= 0.002.

## Discussion

The IMPACT-HAP project demonstrated the feasibility of developing a performance improvement project for HAP, VAP, and HCAP based on the ATS/IDSA guidelines [1], while operationalizing and executing it at the local level in multiple centers. The project included features of other successful critical pathways [8-10], such as appointing leaders to champion the project, actively involving relevant stakeholders, and benchmarking. Initial planning by representatives from each center required three months to evaluate the clinical literature, prepare a consensus pathway based on the ATS/IDSA guidelines [1], and develop a data collection form and Internet-based data repository. The pathway was intentionally flexible to accommodate inter- and intra-center differences. Multidisciplinary teams from each center adapted the empiric therapy recommendations based on their local epidemiology and hospital formulary, another feature of other successful projects [11-14]. Implementation at the local level required approximately six months. Different educational programs were offered to pulmonary/critical care and infectious diseases attending physicians and fellows, house staff, pharmacists, nursing and respiratory therapy staff, including slide presentations for formal grand rounds and lectures -- all of which were supplemented with printed materials to lead clinicians through the process for evaluating suspected nosocomial pneumonia, assessing signs and symptoms, prescribing empiric antibiotics, de-escalating antibiotics, and stopping therapy.

We had initially planned to complete our educational series at the end of the implementation period in order to assess the impact of our initiative. Upon implementation, however, we recognized the need for continual education to accommodate monthly rotations by house staff, some of whom had not attended the formal grand rounds or lectures. This is not unexpected, as recurrent education is yet another feature of other successful interventions [8,9,15,16]. This approach, however, has implications for assessing compliance with quality performance indicators and may have confounded our ability to detect differences between pre- and post-implementation periods.

Compliance with the quality performance indicators related to making the clinical and microbiologic diagnosis of HAP, VAP, and HCAP (QI-1 and QI-2) exceeded 80%. Compliance was high before implementation of our project and remained high during post-implementation. These findings are consistent with the aggressive management of critically ill patients, the high quality of care generally practiced in ICUs, and the knowledge base at these four academic centers.

We found a statistically significant improvement in administration of initial empiric therapy compliant with ATS/IDSA guidelines (QI-3: pre- vs post-implementation, 30.7% vs 43.7%; *P *= 0.01). Notably, improvement became evident during implementation and, importantly, was sustained throughout post-implementation. The most common reason for noncompliance was failure to administer a secondary gram-negative agent, which is consistent with the selective implementation of the consensus pathway at participating centers in the IMPACT-HAP study. Specifically, two centers did not use the secondary gram-negative agent because their local antibiograms indicated adequate coverage with the primary gram-negative agent or reserved the secondary gram-negative agent for patients with VAP. Therefore, initial empiric therapy could be compliant with center-specific guidelines but not with ATS/IDSA guidelines. Others have also questioned the need for dual gram-negative coverage in patients with risk factors for MDROs because of concerns regarding potential renal toxicity associated with aminoglycoside use and lack of an additional benefit based on the local microbiology [17]. A recent survey of more than 800 physicians provides further insight [18]. Although 71% were aware of published guidelines, participants chose guideline-concordant antibiotic regimens for HCAP only 9% of the time. Consistent with our experience, participants failed to choose a secondary antipseudomonal agent for HCAP up to 36% of the time [18].

The next two quality performance indicators addressed measures to avoid excessive antibiotic use. Compliance with short-course therapy doubled during post-implementation (QI-4: 25.0% vs 52.9%); however, this difference was not statistically significant and less than 10% of our patients met the criteria for short-course therapy. Antibiotics were de-escalated on Day 3 based on microbiologic findings only in about one-third of candidates for de-escalation during both periods (QI-5b: 32.4% vs 37.5%). We did not capture reasons for failure to de-escalate, which merits further evaluation as this is an additional area for potential improvement. The last quality performance indicator, clinical success at Day 14, remained unchanged (QI-6, 68.0% vs 66.4%). Potential reasons for the inability to show changes in clinical outcomes despite improvement in compliance with empiric therapy include early adoption of the protocol during the implementation phase and the decision to continue educational efforts throughout the study despite our initial plans to complete our educational series at the end of the implementation phase, as previously discussed. Another consideration is the high clinical success rate observed during the pre-implementation period (reasonable for critically ill patients in the ICU with HAP, VAP, and HCAP), making it difficult to demonstrate further improvement.

While the compliance rates for certain quality performance indicators including compliance with empiric antibiotics appear low, our collective compliance rates were generally consistent with those in studies comparing outcomes before and after implementation of guidelines for severe HAP and VAP [12,13,19] or studies validating guidelines for HAP in the ICU [20-22]. Previous studies had several limitations, such as being conducted at single centers and usually having limited numbers of patients, although Dellit *et al. *evaluated 819 patients [19]. The three validation studies [20-22] did not include pre-implementation data. Most studies evaluated the older 1996 ATS guidelines [23] or center-specific guidelines [13,19,22]; only Ferrer *et al. *[20] evaluated the 2005 ATS/IDSA guidelines [1]. Compliance rates were variable partly because of differences in study methods. In validation studies, compliance rates for empiric therapy were 49% [21] and 58% [20]. In the third validation study [22], overall compliance with standard operating procedures (SOPs) was reported as either higher or lower than 70%. Only 34% of patients were in the high compliance group [22]. Compliance with empiric antibiotics was not reported in the other studies [12,13,19].

Further studies are needed to validate the ATS/IDSA guidelines for patients with HAP, HCAP, and VAP. Some studies have shown that compliance with guidelines shortens the duration of mechanical ventilation and ICU stay [22] or is associated with a higher percentage of adequate antibiotics, which in turn leads to reduced mortality [12]. In another study of HCAP, receipt of empiric therapy not recommended by guidelines was independently associated with mortality after adjusting for other variables [24]. Most studies, however, fail to demonstrate significant correlations between compliance and outcomes [13,19-21]. Our findings on the relationships between compliance and outcomes are reported separately [25]. Additional clinical findings from IMPACT-HAP, such as a new score to predict mortality [26] and the relationship of vancomycin minimal inhibitory concentration (MIC) to mortality [27], are also reported separately.

Our project had several limitations. Most importantly, the participants were adults in multiple ICUs, an inherently complex group with many comorbidities. This was a non-randomized, observational study with the natural limitations of real-world academic medical center practices. However, data from IMPACT-HAP were for the most part prospectively collected and validated before entry into the data repository. Another limitation was the inability to capture all patients admitted to these ICUs during the study period. Specifically, we enrolled as many patients as possible but missed some, especially those admitted on weekends or discharged from the ICU before we could learn of them. Furthermore, patient enrollment was not evenly distributed among centers. For these reasons, our findings may not be generalizable to other ICU populations or patients treated outside the ICU setting. Finally, feedback was not provided to investigators until after the post-implementation period was completed. Real-time feedback might have improved compliance with ATS/IDSA guidelines and other quality indicators.

## Conclusions

Developing a multi-center performance improvement project for implementation of the ATS/IDSA guidelines for HAP, HCAP, and VAP is feasible. Important features of IMPACT-HAP included flexibility to accommodate expected differences in unit-specific epidemiologic data and hospital formularies, ongoing education, and benchmarking. Diagnostic work-ups were performed according to ATS/IDSA guidelines for most patients. Empiric antibiotics were compliant with the guidelines in less than half of the patients; however, improvement in compliance during post-implementation was statistically significant and was sustained. Similarly, short-course therapy and de-escalation were performed in no more than half of eligible patients. The low overall performance in these areas despite educational interventions suggests the need for additional studies to better understand how to influence physician behavior. Additional studies are also needed to validate ATS/IDSA guidelines for HAP, HCAP, and VAP.

## Key messages

• A performance improvement project for implementing ATS/IDSA guidelines for HAP, VAP, and HCAP should be flexible enough to accommodate local epidemiology and hospital formulary considerations.

• The project should include leaders who can champion the initiative and should engage relevant stakeholders.

• Educational efforts should be repeated routinely and continued indefinitely in training centers.

• Benchmarking should be performed to provide feedback to participating centers.

## Abbreviations

APACHE: Acute Physiology and Chronic Health Evaluation; ATS: American Thoracic Society; CPIS: clinical pulmonary infection score; HAP: hospital-acquired pneumonia; HCAP: healthcare-associated pneumonia; IDSA: Infectious Diseases Society of America; IMPACT-HAP: Improving Medicine Through Pathway Assessment of Critical Therapy in Hospital-Acquired Pneumonia; MDRO: multidrug resistant organisms; MIC: minimal inhibitory concentration; MRSA: methicillin-resistant *Staphylococcus aureus*; QI: quality indicator; SOPs: standard operating procedures; VAP: ventilator-associated pneumonia.

## Competing interests

Funding for this study was provided by Pfizer Inc., US Medical. The University of Louisville Foundation was responsible for project oversight and distribution of funds to participating institutions. JEM has served on advisory boards for Madcat Healthcare, Pfizer, Astellas, and Merck; and received educational grants from Fallon Medica. DHK has received research support from Pfizer, and has served as a consultant to and is on the speakers' bureaus of Astellas, Cubist, Glaxo Smith Kline, and Pfizer. MJZ has received honoraria for lectures from Pfizer, Cubist, and Astellas, as well as grant support from Henry Ford Hospital, Pfizer, Johnson and Johnson, and Cubist. JAR has received research support from Pfizer, is a consultant for Pfizer, and has received honoraria from Pfizer, Merck, and Wyeth for lectures. PP has no conflicts of interest to disclose. VLW, KDF and EGS are employees of Pfizer, Inc.

## Authors' contributions

JEM, DHK, MJZ, JAR, PP, EGS and KDF contributed to project development and implementation. JAR, PP and VLW had full access to all data and take responsibility for the integrity of data and accuracy of data analysis. All authors contributed to analysis and interpretation of data, and to drafting of the manuscript and critical revision for important intellectual content. All authors read and approved the final manuscript.

## Supplementary Material

Additional file 1 **Center-specific algorithm**. Click here for file
