# Supplementary material for: Development and implementation of a performance improvement project in adult intensive care units: overview of the Improving Medicine Through Pathway Assessment of Critical Therapy in Hospital-Acquired Pneumonia (IMPACT-HAP) study
Source: Crit Care. 2011 Jan 25;15(1):R38. doi: 10.1186/cc9988 (PMC3222076; doi:10.1186/cc9988)
Supplement: Additional file 1 — Center-specific algorithm. [file cc9988-S1.DOC]

Additional file 1. Center-Specific Algorithm
